# The effect of mid-life insulin resistance and type 2 diabetes on older-age cognitive state: the explanatory role of early-life advantage

**DOI:** 10.1007/s00125-019-4949-3

**Published:** 2019-07-29

**Authors:** Sarah-Naomi James, Andrew Wong, Therese Tillin, Rebecca Hardy, Nishi Chaturvedi, Marcus Richards

**Affiliations:** MRC Unit for Lifelong Health and Ageing at UCL, University College London, 1–19 Torrington Place, London, WC1E 7HB UK

**Keywords:** Cognitive ageing, Cognitive function, Insulin resistance, Life course, Older age, Type 2 diabetes

## Abstract

**Aims/hypothesis:**

Type 2 diabetes, hyperglycaemia and insulin resistance are associated with cognitive impairment and dementia, but causal inference studies using Mendelian randomisation do not confirm this. We hypothesised that early-life cognition and social/educational advantage may confound the relationship.

**Methods:**

From the population-based British 1946 birth cohort, a maximum number of 1780 participants had metabolic variables (type 2 diabetes, insulin resistance [HOMA2-IR] and HbA_1c_) assessed at age 60–64 years, and cognitive state (Addenbrooke’s Cognitive Examination III [ACE-III]) and verbal memory assessed at age 69 years. Earlier-life measures included socioeconomic position (SEP), cognition at age 8 years and educational attainment. Polygenic risk scores (PRSs) for type 2 diabetes were calculated. We first used a PRS approach with multivariable linear regression to estimate associations between PRSs and metabolic traits and later-life cognitive state. Second, using a path model approach, we estimated the interrelationships between earlier-life measures, features of mid-life type 2 diabetes and cognitive state at age 69 years. All models were adjusted for sex.

**Results:**

The externally weighted PRS for type 2 diabetes was associated with mid-life metabolic traits (e.g. HOMA2-IR *β* = 0.08 [95% CI 0.02, 0.16]), but not with ACE-III (*β* = 0.04 [−0.02, 0.90]) or other cognitive outcomes. While there was an association between HOMA2-IR and subsequent ACE-III (*β* = −0.09 [−0.15, −0.03]), path modelling showed no direct effect (*β* = −0.01 [−0.06, 0.03]) after accounting for the association between childhood SEP and education with HOMA2-IR. The same pattern was observed for later-life verbal memory.

**Conclusions/interpretation:**

Associations between type 2 diabetes and mid-life metabolic traits with subsequent cognitive state do not appear causal, and instead they may be explained by SEP in early life, childhood cognition and educational attainment. Therefore, glucose-lowering medication may be unlikely to combat cognitive impairment in older age.

**Electronic supplementary material:**

The online version of this article (10.1007/s00125-019-4949-3) contains peer-reviewed but unedited supplementary material, which is available to authorised users.

## Introduction

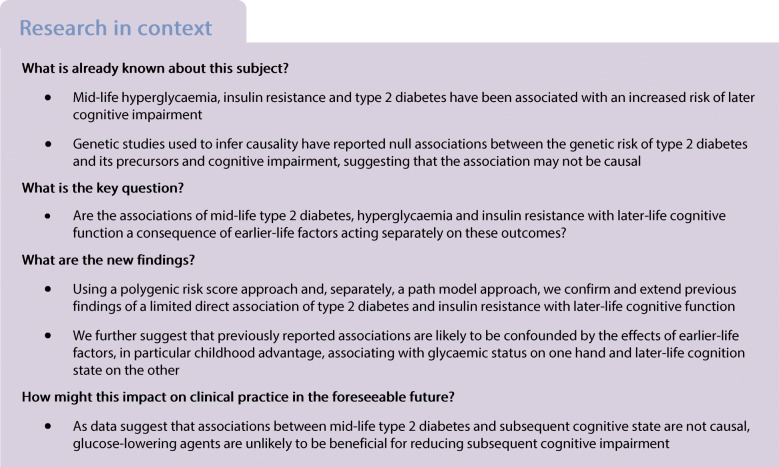



Cross-sectional and longitudinal studies have demonstrated associations between mid-life hyperglycaemia, insulin resistance and type 2 diabetes and increased risk of cognitive impairment, Alzheimer’s disease and all cause dementia [1–13]. Despite the major public health implications of this link, the underlying pathways remain poorly understood [14]. However, genetic studies—more specifically, Mendelian randomisation studies, which use genetic predictors of diabetes as unconfounded instruments to directly assess causality—have reported null associations between the genetic risk of type 2 diabetes and cognitive ability [15] and later-life cognitive impairment [15, 16], and between the genetic risk of type 2 diabetes, fasting glucose and insulin resistance and all-cause dementia and Alzheimer’s disease [17, 18]. These compelling yet contradictory findings suggest that the relationship between type 2 diabetes and its associated features and later-life cognitive impairment may not be directly causal, and that other pathways related to type 2 diabetes, or processes occurring as a comorbidity or result of diabetes and its dysmetabolic precursors, may be aetiologically important in contributing to cognitive impairment/dementia risk.

The life course factors of childhood socioeconomic position (SEP), childhood cognitive ability and educational attainment are associated with mid-life type 2 diabetes risk [19] and, in separate studies, later-life cognitive function [17, 20]. We therefore hypothesise that the association between mid-life type 2 diabetes, hyperglycaemia and insulin resistance and later-life cognitive function in a prospective analysis may be a consequence of these life course factors acting separately on these outcomes and subsequently confounding the relationship. To our knowledge, these genetic and life course relationships have not yet been studied comprehensively together.

We therefore aimed, first, to examine the relationship between mid-life type 2 diabetes, hyperglycaemia and insulin resistance, and the polygenic risk scores (PRSs) for these traits, and later-life cognitive state (Addenbrooke’s Cognitive Examination III [ACE-III]) and memory. Second, we aimed to estimate the independent effects of early-life factors on mid-life type 2 diabetes features and later-life cognitive state by testing a path model incorporating earlier-life factors (father’s social class, childhood cognitive ability, educational attainment) to mid-life type 2 diabetes (and hyperglycaemia and insulin resistance) to later-life cognitive state and memory.

## Methods

### Participants

**Fig. 1:**
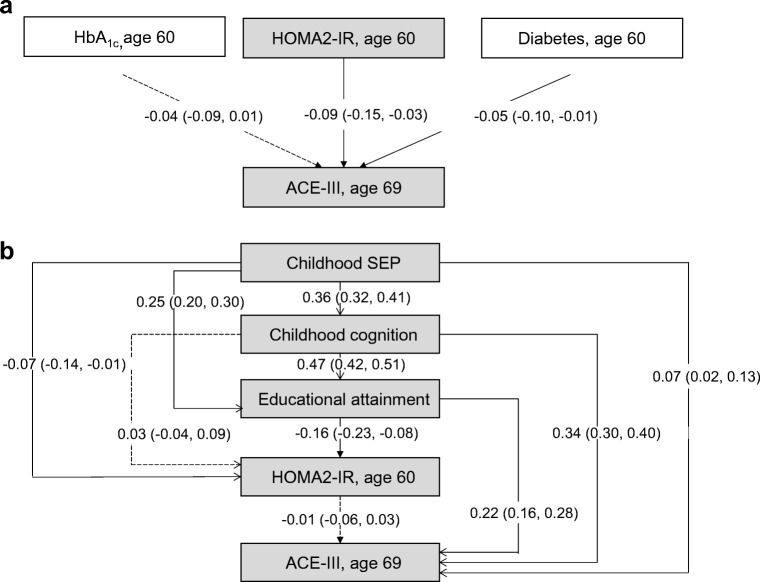
(**a**) Simple path model for the association of mid-life HbA_1c_, HOMA2-IR and type 2 diabetes with later-life cognitive state (assessed using ACE-III), adjusted for sex. HOMA2-IR showed the strongest association and was selected for further modelling. (**b**) Path model for ACE-III in relation to childhood SEP, childhood cognition and educational attainment, together with mid-life HOMA2-IR. All path *β* coefficients are standardised and are mutually adjusted and additionally adjusted for sex. Dashed lines represent non-significant paths at the 5% level (*p* > 0.05); *n* = 1379

The Medical Research Council (MRC) National Survey of Health and Development (NSHD, also known as the British 1946 birth cohort) recruited a representative sample of 5362 men and women born in England, Scotland and Wales in 1 week in March 1946 [21]. It is the oldest British birth cohort with repeated data collected since birth. The most recent data collection, the 24th, was conducted between 2014 and 2015, when participants were aged 68–69 years [22]. At age 69 years, following a postal questionnaire at age 68 years, participants still alive and with a known current address in mainland Britain (*n* = 2698) were invited to have a home visit; 2149 (80%) completed a visit. More detailed information and a flow diagram about the follow-up rates and attrition of participants at the latest stage of recruitment is given in Kuh et al (2016) [22] and electronic supplementary material (ESM) Fig. 1. For the most recent data collection, we obtained ethical approval from Queen Square Research Ethics Committee (REC) (14/LO/1073) and Scotland A REC (14/SS/1009). All participants gave written informed consent.

### Cognitive outcomes

The ACE-III, a test of cognitive state [23], was used as a primary outcome measure at age 69 years. The ACE-III is divided into five domains: attention and orientation (scored 0–18); verbal fluency (0–14); memory (0–26); language (0–26); and visuospatial function (0–16). Thus, the maximum total score is 100. A customised version of the ACE-III was administered by iPad using ACEMobile (www.acemobile.org, version 1, accessed 1 January 2015); where this was not possible, a paper version was used. All offline scoring was undertaken by trained personnel. Of the 2149 participants with a home visit at age 69 years, 32 refused or were unable to undertake the ACE-III. Of the remaining 2117, 35 undertook it but did not complete it and data from 353 participants were corrupt through equipment failure. Thus, complete ACE-III data were available for 1729 participants, 80% of those who received home visit (ESM Fig. 1). For those interviewed at age 69 years, there were no statistical differences in variables included in this analysis for those with and without missing ACE-III data (data not shown).

Verbal memory was tested at age 69 years using a 15 item word-learning task; each word was shown for 2 s. When all words were shown, the study member was asked to write down as many words as possible. This was performed over three identical trials, and the total number of words correctly recalled was summed (maximum score = 45).

### Type 2 diabetes, HbA_1c_ and insulin resistance

Known type 2 diabetes status was based on self-reports of doctor-diagnosed type 2 diabetes or use of oral glucose-lowering medication up to age 60–64 years. Previous work in the NSHD shows good validity of self-reported diabetes compared with general practitioner notes [24]. Medication use was recorded at ages 36, 43, 53 and 60–64 years and coded to the British National Formulary [25]. For this analysis, those with known type 1 diabetes were excluded (*n* = 6).

HbA_1c_ was measured in blood taken at age 60–64 years and 69 years by ion exchange HPLC on a Tosoh analyser (Tosoh Bioscience, Tessenderlo, Belgium). A fasting blood sample was collected at age 60–64 years. Samples were analysed for glucose (measured by enzymatic assay using hexokinase coupled to glucose 6-phosphate dehydrogenase, using a Siemens Dimension Xpand analyser, Siemens Medical Solutions, Erlangen, Germany) and insulin (measured by fluoroimmunoassay using a PerkinElmer AutoDELFIA analyser, PerkinElmer, Waltham, MA, USA). HOMA2-IR was calculated [26].

### PRSs of traits

Blood samples from participants at age 53 years were genotyped using MetaboChip, a custom Illumina iSelectarray (San Diego, CA, USA) that includes ~200,000 SNPs and covers the loci identified by genome-wide association studies (GWASs) in cardiometabolic diseases, including rare variants identified by the 1000 Genomes Project [27]. Quality control analysis of genotyped samples has been previously reported [28].

Three PRSs were calculated. A type 2 diabetes PRS has previously been derived for NSHD study members [29]. In brief, a genetic risk score was computed using the published coefficients for 65 SNPs identified by a prior GWAS for type 2 diabetes [30, 31]. We additionally derived a PRS for insulin resistance using 17 previously demonstrated genome-wide significant SNPs [32] and for hyperglycaemia using ten previously demonstrated SNPs [32] using PRSice [33]. This calculates the sum of the number of risk alleles (unweighted score) carried by each person, and weights it based on previously published coefficients (weighted score). As is standard practice, SNPs with a minor allele frequency <0.01 were excluded.

### Earlier-life variables (covariables)

#### Childhood cognitive function

Childhood cognitive function at age 8 years was represented as the sum of four tests of verbal and non-verbal ability devised by the National Foundation for Educational Research [34].

#### Childhood SEP

Childhood SEP was represented by the occupation of the father when study members were aged 11 years; if missing at 11 years, occupation was substituted by the father’s occupation class at ages 4 or 15 years. SEP was coded according to the UK Registrar General into six categories (professional, managerial, intermediate, skilled manual, semi-skilled manual and unskilled). For consistency with the other variables, these were coded so that higher values corresponded to higher positions.

#### Educational attainment

The highest educational attainment or training qualification achieved by 26 years was classified according to the Burnham scale [35] and grouped into the following: no qualification; below ordinary secondary qualifications (e.g. vocational qualifications); ordinary level qualifications (‘O’ levels or their training equivalents); advanced level qualifications (‘A’ levels or their equivalents); or higher education (degree or equivalent).

### Statistical analysis

Individuals were included in the analysis if they had: at least one measure of earlier-life factors (childhood SEP, childhood cognition, education); at least one measure of type 2 diabetes, insulin resistance or hyperglycaemia at age 60–64 years; and at least one measure of cognitive function (ACE-III or verbal memory) at age 69 years.

#### PRS regression models

Using logistic and linear regression models, we first performed a sensitivity check to see whether the PRS for type 2 diabetes was associated with type 2 diabetes, hyperglycaemia and insulin resistance measures in our sample aged 60–64 years. We then investigated the association between genetic risk of type 2 diabetes, insulin resistance and hyperglycaemia with later-life cognition (ACE-III score at age 69 years) and memory (verbal memory at age 69 years) using linear regression models further adjusted for sex.

#### Earlier-life factors, mid-life diabetes and later-life cognitive state

We used path analysis to assess the association between mid-life type 2 diabetes and later-life cognitive state and to assess the relative contributions of earlier life course factors to mid-life type 2 diabetes and to later-life cognitive state. Path analysis is a technique often used in life course epidemiology [36]. It is an extension of regression models, whereby the models would be similar if we were to use simple path modelling of the independent variable on the dependent variable (e.g. type 2 diabetes on ACE-III), yet path analysis can further estimate more complex relationships [37]. It enables the examination of multiple associations simultaneously; in this case, it has been used to estimate regression equations for simultaneous multiple paths between: (1) mid-life type 2 diabetes and cognitive state; (2) earlier-life variables; (3) earlier-life variables and mid-life type 2 diabetes; and (4) earlier-life variables and cognitive state. Path analysis can additionally decompose the total effect of an exposure on an outcome into direct effects (effect of exposure on outcome not mediated through other measures) and indirect effects (effect mediated through other measured risk factors) [20, 36, 38].

This approach is particularly suitable for our research question given that: the earlier-life-course factors are closely related; the use of longitudinal data enables examination of the temporal relationship between variables; and the model is derived from a coherent a priori evidence base [38]. As we are particularly interested in quantifying and estimating the individual coefficients for each path to both mid-life type 2 diabetes and cognitive state, this approach enabled us to distinguish between paths of individual early-life factors to mid-life type 2 diabetes and to later-life cognitive state accordingly. Particular hypotheses include: (1) strong paths from earlier-life factors to later-life cognitive state, and to mid-life type 2 diabetes; and (2) the effects of earlier-life factors on cognitive state are mediated through mid-life type 2 diabetes.

#### Estimating the model

All statistical analyses were conducted using STATA 14.1 (StataCorp, College Station, TX, USA); path modelling was conducted using the ‘sem’ and ‘glm’ package (StataCorp). All models were adjusted for sex and, in line with previous studies, estimated using full information maximum likelihood, which allows for missing data and is preferable to estimation based on complete data.

**Fig. 2:**
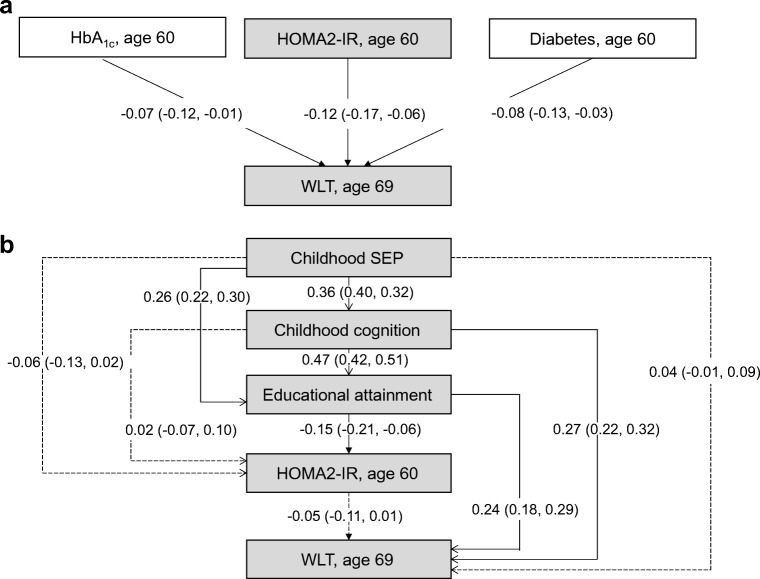
(**a**) Simple path model for the association of mid-life HbA_1c_, HOMA2-IR and type 2 diabetes with later-life verbal memory (assessed using a word-learning task [WLT]), adjusted for sex. HOMA2-IR showed the strongest association and was selected for further modelling. (**b**) Path model for the WLT in relation to childhood SEP, childhood cognition and educational attainment, together with mid-life HOMA2-IR. All path *β* coefficients are standardised and are mutually adjusted and additionally adjusted for sex. Dashed lines represent non-significant paths at the 5% level (*p* > 0.05); *n* = 1379

First, a simple path analysis (similar to a linear regression model adjusted for sex) was used to test associations between mid-life type 2 diabetes, hyperglycaemia and insulin resistance at age 60–64 years and later-life cognitive state at age 69 years (ACE-III score [Fig. 1a]) and verbal memory [Fig. 2a]. To reduce the number of further multiple comparison tests, the feature (HbA_1c_ or HOMA2-IR) with the strongest association and significance with ACE-III scores was included in further statistical modelling. Coefficients were standardised based on available data.

Second, path modelling incorporating all variables of interest, in the pattern laid out in Fig. 1b, were used to estimate interrelationships between earlier-life covariables (childhood cognition, childhood SEP and education), mid-life type 2 diabetes and its features, and later-life cognitive state (Figs 1b and 2b). The numerical values refer to standardised regression weights whereby all paths were mutually adjusted and further adjusted for sex.

In line with existing studies [38], root mean square error of approximation (RMSEA) and comparative fit index (CFI) estimates were used to assess the fit of the model to the data. RMSEA<0.05 and CFI close to one indicates a close fit to the data. Assuming that normally distributed latent variables underlie responses on an ordinal scale [20], estimates are presented as standardised coefficients for observed continuous and ordinal variables (Figs 1, 2).

### Sensitivity analyses

To explore whether the pattern of our results was affected by type 2 diabetes-related medication, we used two approaches. First, for those on diabetes medication, we replaced measured HbA_1c_ and HOMA2-IR scores with the maximum value within the cohort (used for all analyses where they are outcomes and predictors). Second, we re-ran the initial multivariable regression analyses using an additional covariate indicator for people on diabetes medication (*n* = 128) (ESM Fig. 4). Multivariable regression analyses were additionally re-run excluding participants with potentially clinically significant cognitive impairment (81 study members [5%] fell below the clinically validated ACE-III <82 threshold) and adjusting for duration of diabetes (ESM Figs 5, 6).

## Results

Participant characteristics are shown in Table 1. The maximum sample numbers for outcomes using the ACE-III and memory test were *n* = 1494 and *n* = 1780, respectively.

**Table 1:** Characteristics of study participants

Characteristic	Mean (SD)^a^, all	Participants available	
		*n*	%
Sex (male), %	48	1803	100
Higher childhood SEP, %	47	1737	96
Higher educational attainment, %	40	1759	98
Child cognition SD, age 8 years	0.13 (0.8)	1628	90
Known T2DM by age 69 years, *n* (%)	169 (10)	1701	94
HbA_1c_ at age 69 years			
%	5.8 (0.6)	1486	82
mmol/mol	40 (6.6)	1486	82
Ever on diabetes medication, *n* (%)	128 (7.3)	1803	100
Smoking status (never/ex/current), %	30, 61, 8	1782	99
Characteristics at age 69 years			
WHR			
Men	0.96 (0.07)	856	47
Women	0.87 (0.07)	936	52
BMI, kg/m^2^	28.0 (5.1)	1789	99
BP, mmHg			
Systolic	132 (16)	1793	99
Diastolic	73 (10)	1793	99
Heart rate, bpm	68.8 (10.9)	1794	100
Total cholesterol, mmol/l	5.2 (1.2)	1521	84
HDL-cholesterol, mmol/l	1.5 (0.4)	1515	84
LDL-cholesterol, mmol/l	2.9 (0.9)	1499	83
Triacylglycerols, median (IQR), mmol/l	1.5 (1.1–2)	1521	84
Alcohol (≥×4/week), *n* (%)	507 (30)	1789	99
Clinical history, any incidence, *n* (%)			
Stroke	69 (4)	1797	100
Angina	113 (7)	1797	100
Heart attack	65 (4)	1797	100
Heart failure	42 (3)	1797	100
Prior cardiac event	218 (13)	1797	100
*APOE*-*ε4*^b^ (absent, hetero-, homozygous), %	(71, 25, 3)	1582	88
Mid-life T2DM measures at age 60–64 years			
Known T2DM, *n* (%)	101 (7)	1799	100
HbA_1c_			
%	5.8 (0.7)	1671	93
mmol/mol	40 (7.7)	1671	93
HOMA2-IR	0.9 (0.6)	1379	76
Later-life cognitive measures, age 69 years			
ACE-III (max score = 100)	91.5 (5.9)	1494	83
Verbal memory score (max score = 45)	22.3 (6.1)	1780	99

^a^Unless otherwise stated

^b^Encodes apolipoprotein E (ε4 allele)

IQR, interquartile range; T2DM, type 2 diabetes mellitus

### Associations with PRSs

A higher PRS for type 2 diabetes was strongly and significantly associated with a higher likelihood of having known type 2 diabetes by age 60–64 years (OR 1.08, 95% CI 1.03, 1.11) and higher HOMA2-IR and HbA_1c_ levels at age 60–64 years (Table 2). There was little evidence that higher PRSs for type 2 diabetes, insulin resistance or hyperglycaemia were associated with lower ACE-III scores at age 69 years or lower verbal memory; the direction of regression coefficients suggested that higher risk scores were associated with lower cognitive state, though associations were weak and not significant at the 5% level (Table 2).

**Table 2:** Regression analyses between PRSs for type 2 diabetes/insulin resistance/hyperglycaemia with later-life cognitive outcomes

PRS exposure	Unweighted PRS			Externally weighted PRS		
	*β*/OR^a^	*p*	95% CI	*β*/OR^a^	*p*	95% CI
Type 2 diabetes PRS predictor						
Known T2DM at age 60–64 years^a^	1.08	<0.001	1.03, 1.11	1.45	<0.01	1.10, 1.84
HOMA2-IR at age 60–64 years	0.11	<0.001	0.01, 0.16	0.08	0.01	0.02, 0.16
HbA_1c_ at age 60–64 years	0.12	<0.001	0.02, 0.17	0.14	<0.001	0.11, 0.28
ACE-III at age 69 years	0.02	0.39	−0.03, 0.08	0.04	0.15	−0.02, 0.90
Verbal memory at age 69 years	−0.01	0.72	−0.07, 0.05	0.001	0.96	−0.38, 0.36
Insulin resistance PRS predictor						
ACE-III at age 69 years	0.04	0.25	−0.07, 0.09	0.03	0.12	−0.04, 0.56
Verbal memory at age 69 years	−0.01	0.83	−0.08, 0.09	−0.01	0.81	−0.39, 0.24
Hyperglycaemia PRS predictor						
ACE-III at age 69 years	0.02	0.42	−0.04, 0.05	0.03	0.24	−0.07, 0.41
Verbal memory at age 69 years	−0.001	0.79	−0.13, 0.12	−0.01	0.81	−0.51, 0.19

^a^Estimates are *β* coefficients from linear regression models for continuous outcomes (all outcomes except T2DM), and ORs from logistic regression models for dichotomous outcomes (T2DM). T2DM, type 2 diabetes mellitus

### Path modelling

Figure 1 a shows simple path models between mid-life diabetes and its features and the ACE-III scores, adjusting for sex (similar to linear regression models adjusted for sex). Higher HOMA2-IR and known type 2 diabetes status at age 60–64 years were significantly associated with lower ACE-III scores (Fig. 1a). HOMA2-IR was the type 2 diabetes feature with the strongest coefficient with lower ACE-III scores (*β* = −0.09, 95% CI −0.15, −0.03) and lower verbal memory (*β* = −0.12, 95% CI −0.17, −0.06). HOMA2-IR was therefore subsequently selected for further modelling.

Figure 1 b shows life course path models representing associations between all covariables (childhood cognition, childhood SEP and education) and HOMA2-IR and later-life cognitive state. The numerical values refer to standardised regression weights whereby all paths are mutually adjusted and further adjusted for sex. Figure 2 and ESM Fig. 2, respectively, show path models replacing: (1) later-life cognitive state with later-life memory; and (2) HOMA2-IR with mid-life type 2 diabetes. Goodness-of-fit statistics indicated that all models were satisfactory (all models: RMSEA = 0.01, CFI = 1.0).

Path modelling revealed that when earlier covariables were mutually adjusted, the path from HOMA2-IR to the ACE-III was significantly attenuated (unadjusted *β* = −0.09, 95% CI −0.15, −0.03; adjusted *β* = −0.01, 95% CI −0.06, 0.03) (Fig. 1).

Paths from covariables to mid-life HOMA2-IR show mutually independent and significant paths from educational attainment (*β* = −0.16, 95% CI −0.23, −0.08) and childhood SEP (*β* = −0.07, 95% CI −0.14, −0.01) to HOMA2-IR at age 60–64 years, with the former path stronger than the latter (Fig. 1b). Paths from covariables to later-life cognitive state show direct mutually independent and significant paths from childhood SEP, childhood cognition and educational attainment to ACE-III scores at age 69 years (*β* = 0.07, 0.34 and 0.22, respectively), with the strongest path shown by childhood cognition.

Similarly, when verbal memory was the later-life cognitive outcome, the path from HOMA2-IR to memory was significantly attenuated when covariables were modelled (unadjusted *β* = −0.12, 95% CI −0.17, −0.06; adjusted *β* = −0.05, 95% CI −0.11, 0.01) (Fig. 2). The paths revealed a similar pattern, with education the strongest path coefficient to HOMA2-IR and childhood cognition the strongest path to later-life memory.

The pattern of findings was similar when type 2 diabetes was the intermediate mid-life diabetes feature (ESM Figs 2 and 3). The pattern of findings were similar but attenuated slightly when models were estimated adding in a covariate for those on diabetes medication up to age 69 years (ESM Fig. 4). Notably, when the analysis was additionally adjusted for diabetes medication use, the path from educational attainment to HOMA2-IR was largely attenuated (from *β* = −0.16 in the original model to *β* = 0.06 in the adjusted model) and became non-significant; the strongest path to HOMA2-IR in this model was childhood SEP (ESM Fig. 4). The pattern of findings for HOMA2-IR was similar when analyses were re-run excluding those with ACE-III scores <82 and adjusting for duration of diabetes (ESM Figs 5 and 6, respectively).

## Discussion

### Main findings

Our results show that the relationship between mid-life type 2 diabetes, insulin resistance and poorer later-life cognitive function or memory are likely to be confounded by the effects of earlier-life factors, in particular childhood advantage, associating with glycaemic status on one hand and late-life cognitive state on the other. Although it has previously been demonstrated that earlier-life influences, such as education, increase the risk of type 2 diabetes [8] and lower cognitive state and dementia [6], very few studies have investigated these shared influences in combination and how these relate to an association between type 2 diabetes and later-life cognitive state [14].

Using a PRS approach and, separately, a path model approach, we confirm and extend previous findings [15–18] of a limited direct association between type 2 diabetes, hyperglycaemia and insulin resistance and later-life cognitive function. Within the same dataset, however, conventional regression analysis shows a strong association between mid-life type 2 diabetes and poor cognitive state. These discordant findings highlight the confounded nature of the observed association and the importance of considering life course influences. While the null association between metabolic PRS and later cognitive state could additionally be explained by the use of a weak genetic instrument and lack of power, and limitations of path analyses include assumptions about latent variables, directionality and unmeasured confounders, using these differential approaches to address the same underlying question—so called triangulation—helps to consolidate our findings [39]. Path analysis cannot prove causality, but it can be used to test models and disprove a model that postulates causal relationships among variables. Taking into account the strengths and limitations of both approaches, it is striking that both analyses suggest the same message: that there is no causal association between mid-life hyperglycaemia and later-life cognitive function at age 69 years.

In contrast to the null direct association between insulin resistance and subsequent cognitive function, we found that mid-life insulin resistance is associated with educational attainment and, independently, with childhood SEP. Mechanisms may include the adoption of healthier behaviours and, in particular, avoidance of obesity [40]. Further, we found that childhood cognition has a strong and independent effect on both older-age cognitive status and memory, with effect sizes equal to or stronger than those for education. A previous large-scale study associated genetic instruments for educational attainment and cognitive function separately with mid-life cognition: strong associations were observed for each of these with verbal-numeric reasoning, but not memory [16].

Other pathways related to type 2 diabetes or processes occurring as a complication of diabetes and its dysmetabolic precursors, such as small vessel disease and mixed vascular and neurodegeneration pathologies, may be aetiologically important in contributing to cognitive impairment/dementia risk [41].

### Strengths and limitations

The major strengths of this study include the unique resource of the NSHD cohort, with: direct measurement of life course factors, including the rarely available item childhood cognition, in a population-representative sample; and repeated collection of data on older-age cognitive state and glycaemic traits. In addition, we have sought to triangulate our results, taking into account the strengths and limitations of differential methods of PRS and path analysis.

Limitations include the relatively small sample size, precluding a formal Mendelian randomisation analysis, and differential loss to follow-up of those who were socioeconomically disadvantaged, which may result in an underestimation of the strength of associations between measures of childhood advantage and cognitive outcomes. In addition, there are relatively few people with diabetes, and HbA_1c_, HOMA2-IR and cognitive state are mostly in a healthy range in this cohort at this age; subsequently, associations observed are expected to be weaker than they would be in more unhealthy ranges. We cannot wholly rule out reverse causality, i.e. that cognitive impairment may result in worse diabetes management. However, we also explored associations across the spectrum of insulin resistance and hyperglycaemia, where such a bias is less likely to be a problem. In addition, our path model analyses assumed that a normally distributed latent variable underlies responses of the ordinal scales used in the analyses (SEP and educational attainment) [20]. Comparisons between path estimates should be considered with this in mind.

Overall, our findings indicate that while mid-life type 2 diabetes and insulin resistance are associated with poorer later-life cognitive state, this association is, in part if not in full, confounded by earlier factors and is not consistent with a direct causal pathway of type 2 diabetes features per se to later-life lower cognitive state. Importantly, we also show that early-life measures of advantage, specifically education and cognition, appear to have independent effects on both older-age metabolic disease and cognitive state. These findings have important implications for developing interventions to reduce the risk of cognitive impairment. Despite efforts, and some promising findings [42], glucose-lowering agents are unlikely to be beneficial. Mechanisms accounting for the dual positive effects of prior advantage on mid-life insulin resistance and later-life cognitive state are not well understood and require further investigation.

## Electronic supplementary material

ESM (PDF 289 kb)

## Data Availability

The data used in this publication are available to bona fide researchers on request to the NSHD Data Sharing Committee via a standard application procedure. Further details can be found at www.nshd.mrc.ac.uk/data (10.5522/NSHD/Q102; 10.5522/NSHD/Q103).

## Abbreviations

ACE-III: Addenbrooke’s Cognitive Examination III
CFI: Comparative fit index
GWAS: Genome-wide association study
MRC: Medical Research Council
NSHD: National Survey of Health and Development
PRS: Polygenic risk score
RMSEA: Root mean square error of approximation
SEP: Socioeconomic position
