# Supplementary material for: The effect of mid-life insulin resistance and type 2 diabetes on older-age cognitive state: the explanatory role of early-life advantage
Source: Diabetologia. 2019 Jul 29;62(10):1891–900. doi: 10.1007/s00125-019-4949-3 (PMC6731197; doi:10.1007/s00125-019-4949-3)

Electronic Supplementary Material (ESM)

ESM Fig. 1: Flow diagram of target samples for the postal questionnaire at age 68 and home visit at age 69 (amended from Kuh et al. 2017).[1]

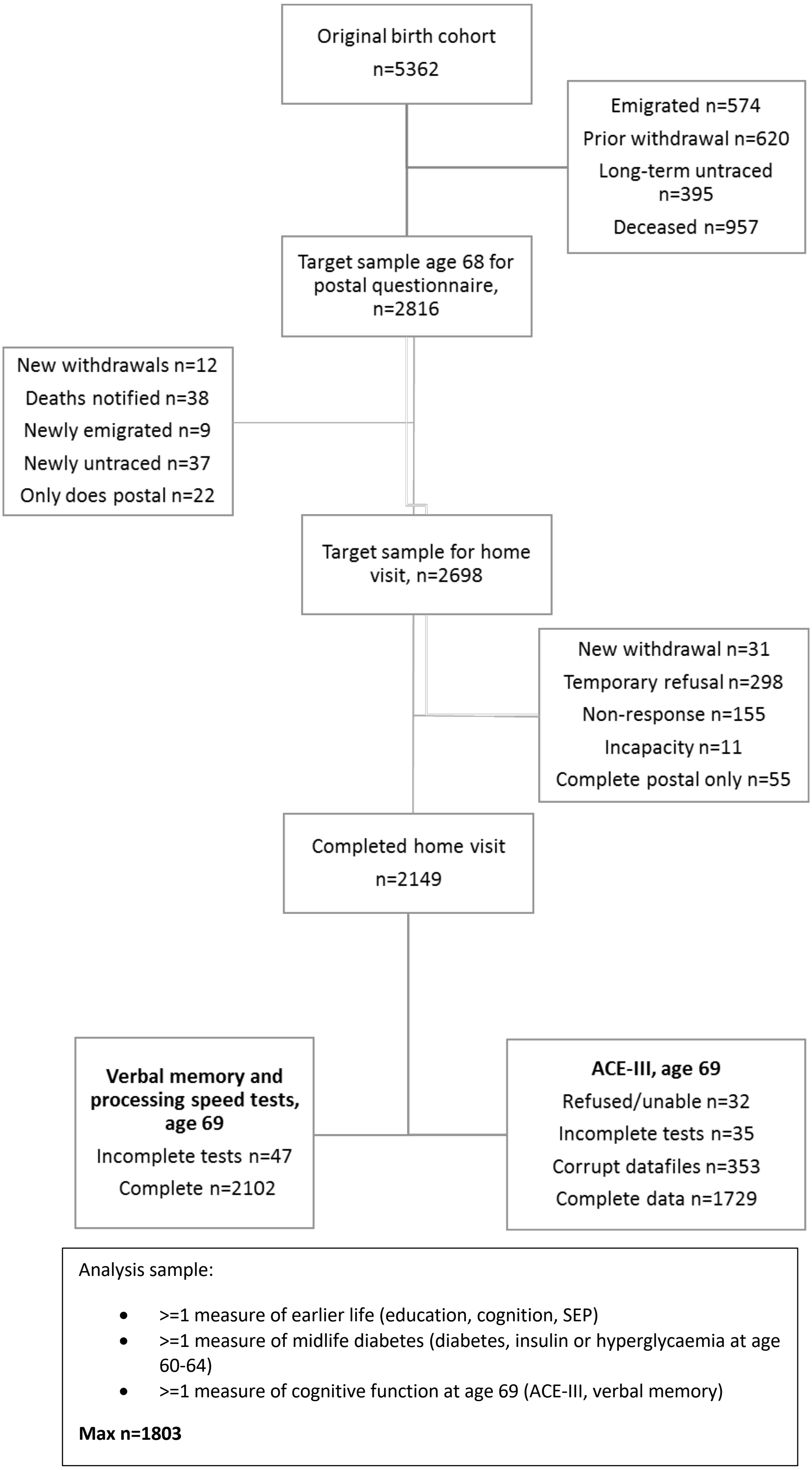

ESM Fig. 2: A) Simple path models for midlife type 2 diabetes to later-life ACE-III B) Path model for the ACE-III in relation to childhood cognition, childhood SEP, educational attainment; with midlife type 2 diabetes. All path coefficients are standardized and are mutually independent and adjusted for sex. Dashed lines represent non-significant paths at the 5% level (p>0.05).

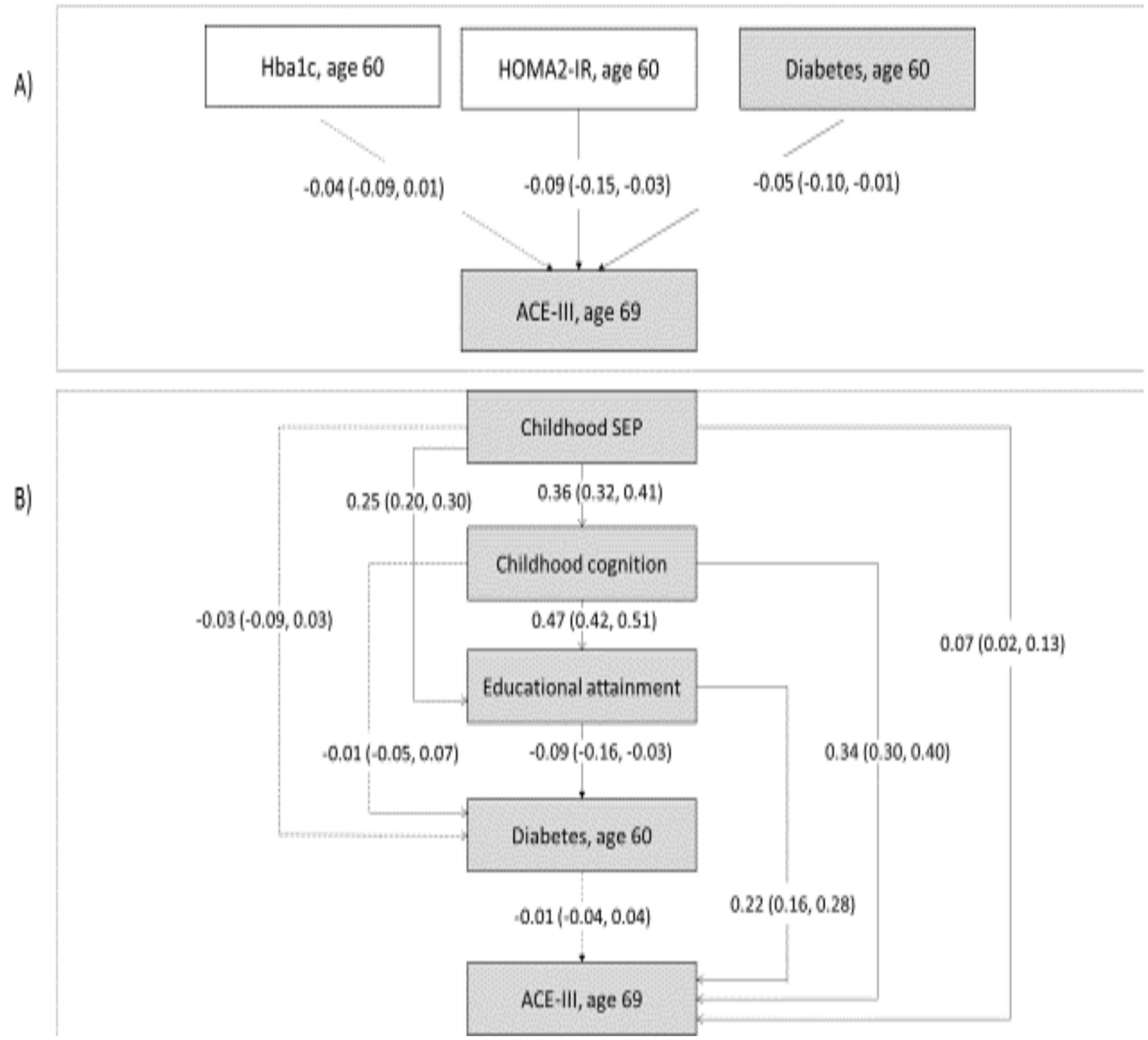

ESM Fig. 3: A) Simple path models for sex-adjusted coefficients from midlife type 2 diabetes to later-life word learning test (WLT, memory) B) Path model for the WLT in relation to childhood cognition, childhood SEP, educational attainment; with midlife type 2 diabetes. All path coefficients are standardized and are mutually independent and adjusted for sex. Dashed lines represent non-significant paths at the 5% level (p>0.05).

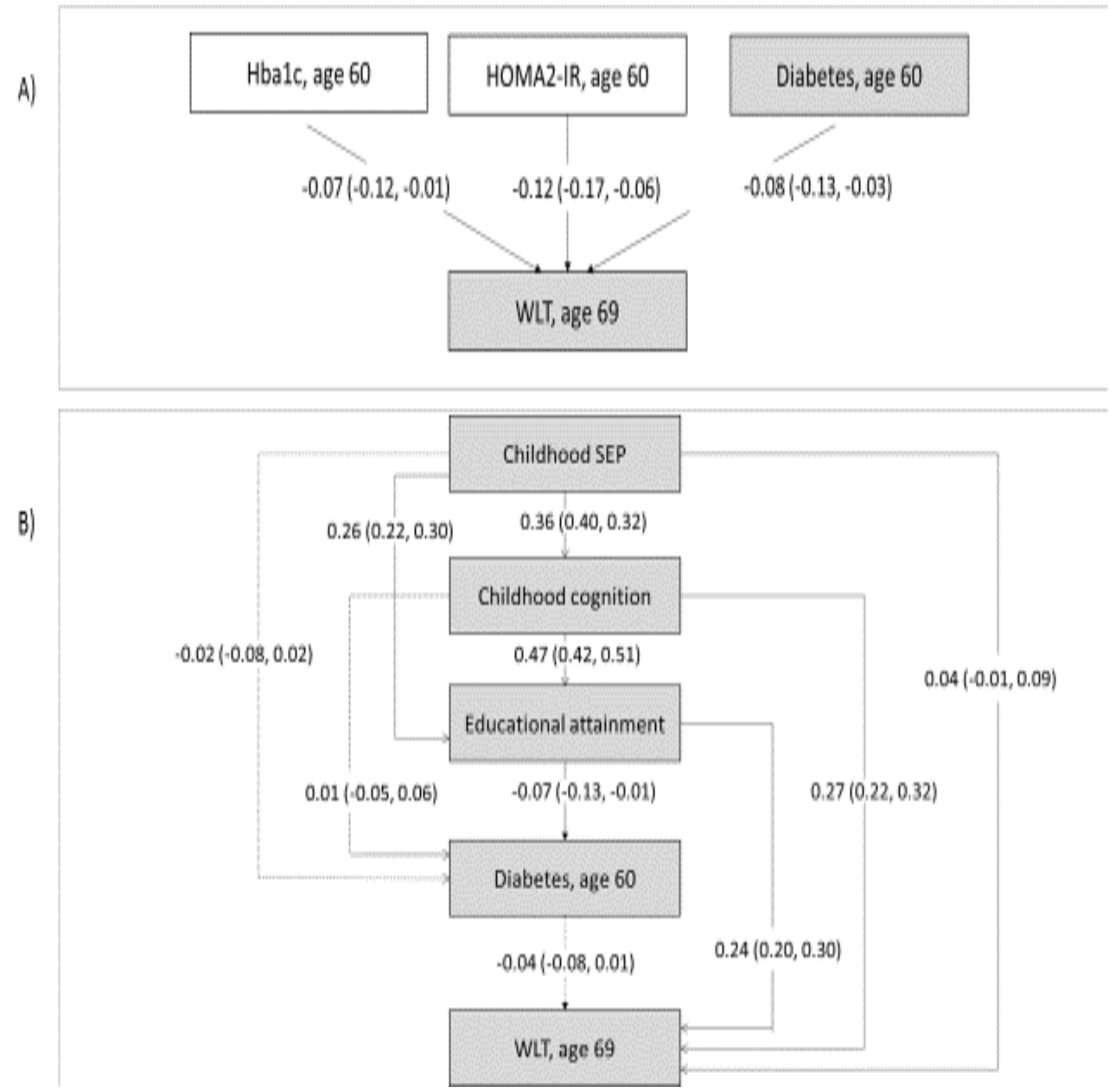

ESM Fig. 4: A) Simple path models for HOMA2-IR to later-life ACE-III (cognitive state) B) Path model for the ACE-III in relation to childhood cognition, childhood SEP, educational attainment; with midlife HOMA2-IR. All path coefficients are standardized and are mutually independent and adjusted for sex and medication use. Dashed lines represent non-significant paths at the 5% level (p>0.05).

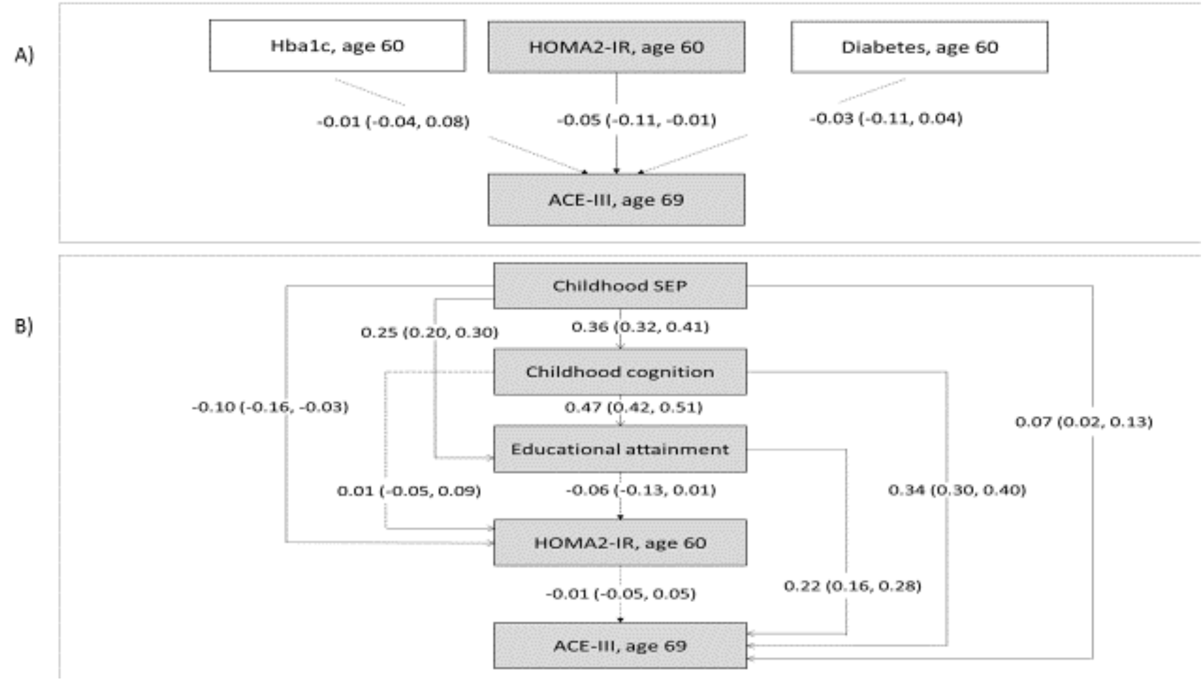

ESM Fig. 5: A) Simple path models for HOMA2-IR to later-life ACE-III (cognitive state) B) Path model for the ACE-III in relation to childhood cognition, childhood SEP, educational attainment; with midlife HOMA2-IR. All path coefficients are standardized and are mutually independent and adjusted for sex and excluding those with ACE-III scores<82. Dashed lines represent non-significant paths at the 5% level (p>0.05).

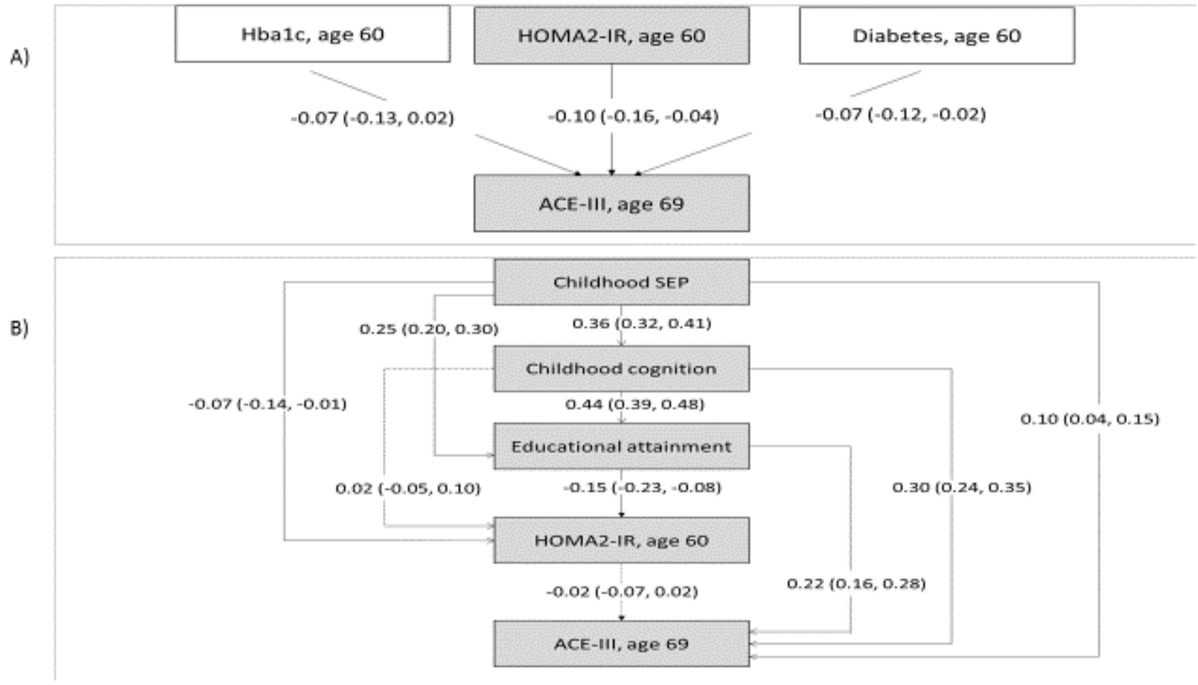

ESM Fig. 6: A) Simple path models for HOMA2-IR to later-life ACE-III (cognitive state) B) Path model for the ACE-III in relation to childhood cognition, childhood SEP, educational attainment; with midlife HOMA2-IR. All path coefficients are standardized and are mutually independent and adjusted for sex and duration of type 2 diabetes. Dashed lines represent non-significant paths at the 5% level (p>0.05).

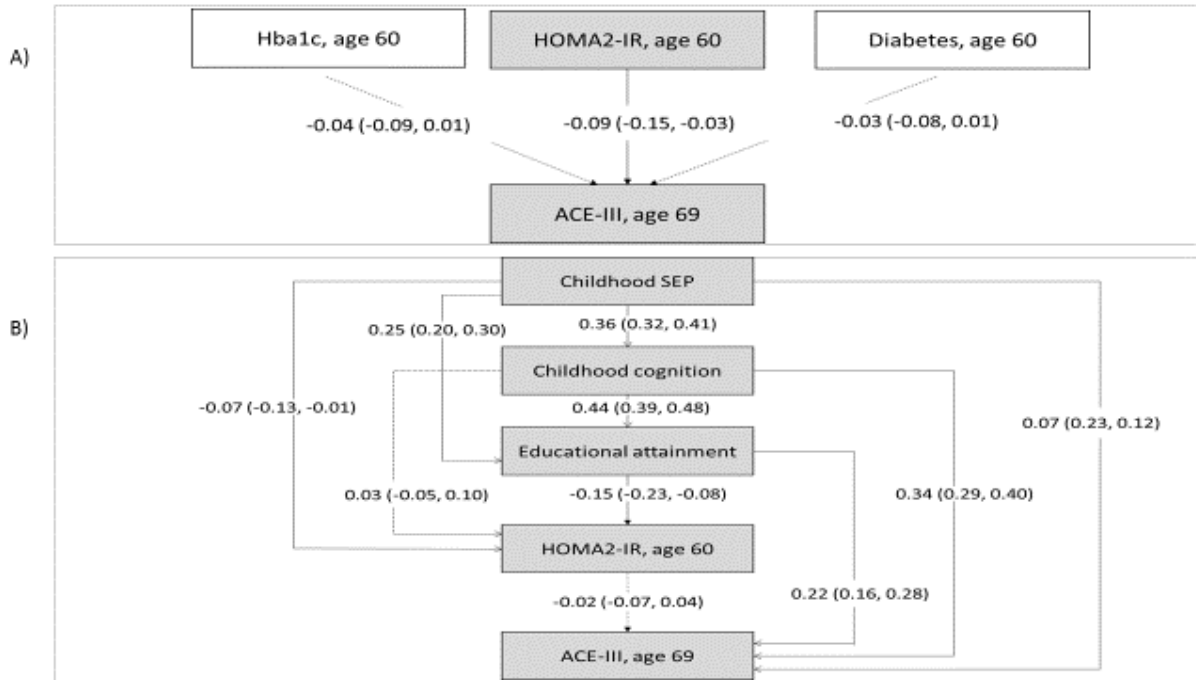

Supplement: Supplementary file 1 — (PDF 289 kb) [file 125_2019_4949_MOESM1_ESM.pdf]
